# SARS-CoV-2 Vaccine Immunogenicity in Patients with Gastrointestinal Cancer Receiving Systemic Anti-Cancer Therapy

**DOI:** 10.1093/oncolo/oyac230

**Published:** 2022-11-07

**Authors:** David K Lau, Maria Aresu, Timothy Planche, Amina Tran, Retchel Lazaro-Alcausi, Julie Duncan, Shannon Kidd, Susan Cromarty, Ruwaida Begum, Isma Rana, Su Li, Ali Abdulnabi Suwaidan, Irene Monahan, David J Clark, Nicholas Eckersley, Henry M Staines, Elisabetta Groppelli, Sanjeev Krishna, Martin Mayora-Neto, Nigel Temperton, Charlotte Fribbens, David Watkins, Naureen Starling, Ian Chau, David Cunningham, Sheela Rao

**Affiliations:** Gastrointestinal and Lymphoma Unit, Royal Marsden NHS Foundation Trust, London and Surrey, UK; Department of Clinical Research and Development, Royal Marsden NHS Foundation Trust, London and Surrey, UK; Centre for Diagnostics & Antimicrobial Resistance, Clinical Academic Group in Institute for Infection & Immunity, St George’s University of London, London, UK; St George’s University Hospitals NHS Foundation Trust, London, UK; Department of Clinical Research and Development, Royal Marsden NHS Foundation Trust, London and Surrey, UK; Gastrointestinal and Lymphoma Unit, Royal Marsden NHS Foundation Trust, London and Surrey, UK; Gastrointestinal and Lymphoma Unit, Royal Marsden NHS Foundation Trust, London and Surrey, UK; Gastrointestinal and Lymphoma Unit, Royal Marsden NHS Foundation Trust, London and Surrey, UK; Gastrointestinal and Lymphoma Unit, Royal Marsden NHS Foundation Trust, London and Surrey, UK; Gastrointestinal and Lymphoma Unit, Royal Marsden NHS Foundation Trust, London and Surrey, UK; Gastrointestinal and Lymphoma Unit, Royal Marsden NHS Foundation Trust, London and Surrey, UK; Gastrointestinal and Lymphoma Unit, Royal Marsden NHS Foundation Trust, London and Surrey, UK; Gastrointestinal and Lymphoma Unit, Royal Marsden NHS Foundation Trust, London and Surrey, UK; Centre for Diagnostics & Antimicrobial Resistance, Clinical Academic Group in Institute for Infection & Immunity, St George’s University of London, London, UK; Centre for Diagnostics & Antimicrobial Resistance, Clinical Academic Group in Institute for Infection & Immunity, St George’s University of London, London, UK; Centre for Diagnostics & Antimicrobial Resistance, Clinical Academic Group in Institute for Infection & Immunity, St George’s University of London, London, UK; Centre for Diagnostics & Antimicrobial Resistance, Clinical Academic Group in Institute for Infection & Immunity, St George’s University of London, London, UK; Centre for Diagnostics & Antimicrobial Resistance, Clinical Academic Group in Institute for Infection & Immunity, St George’s University of London, London, UK; Centre for Diagnostics & Antimicrobial Resistance, Clinical Academic Group in Institute for Infection & Immunity, St George’s University of London, London, UK; St George’s University Hospitals NHS Foundation Trust, London, UK; Institut für Tropenmedizin, Universitätsklinikum Tübingen, Tübingen, Germany; Centre de Recherches Médicales de Lambaréné, Gabon, Lambaréné; Viral Pseudotype Unit (VPU Kent), Medway School of Pharmacy, University of Kent and Greenwich at Medway, Chatham Maritime, Kent, UK; Viral Pseudotype Unit (VPU Kent), Medway School of Pharmacy, University of Kent and Greenwich at Medway, Chatham Maritime, Kent, UK; Gastrointestinal and Lymphoma Unit, Royal Marsden NHS Foundation Trust, London and Surrey, UK; Gastrointestinal and Lymphoma Unit, Royal Marsden NHS Foundation Trust, London and Surrey, UK; Gastrointestinal and Lymphoma Unit, Royal Marsden NHS Foundation Trust, London and Surrey, UK; Gastrointestinal and Lymphoma Unit, Royal Marsden NHS Foundation Trust, London and Surrey, UK; Gastrointestinal and Lymphoma Unit, Royal Marsden NHS Foundation Trust, London and Surrey, UK; Gastrointestinal and Lymphoma Unit, Royal Marsden NHS Foundation Trust, London and Surrey, UK

**Keywords:** SARS-CoV-2, vaccines, COVID-19, gastrointestinal cancer, pseudovirus, anti-spike, immunity, chemotherapy

## Abstract

**Introduction:**

Patients with gastrointestinal (GI) cancers have an increased risk of serious complications and death from SARS-CoV-2 infection. The immunogenicity of vaccines in patients with GI cancers receiving anti-cancer therapies is unclear. We conducted a prospective study to evaluate the prevalence of neutralizing antibodies in a cohort of GI cancer patients receiving chemotherapy following SARS-CoV-2 vaccination.

**Materials and Methods:**

Between September 2020 and April 2021, patients with cancer undergoing chemotherapy were enrolled. At baseline (day 0), days 28, 56, and 84, we assessed serum antibodies to SARS-CoV-2 spike (anti-S) and anti-nucleocapsid (anti-NP) and concomitantly assessed virus neutralization using a pseudovirus neutralization assay. Patients received either the Pfizer/BioNTech BNT162b2, or the Oxford/AstraZeneca ChAdOx1 vaccine.

**Results:**

All 152 patients enrolled had a prior diagnosis of cancer; colorectal (*n* = 80, 52.6%), oesophagogastric (*n* = 38, 25.0%), and hepato pancreatic biliary (*n* = 22, 12.5%). Nearly all were receiving systemic anti-cancer therapy (99.3%). Of the 51 patients who did not receive a vaccination prior to, or during the study, 5 patients had detectable anti-NP antibodies. Ninety-nine patients received at least one dose of vaccine prior to, or during the study. Within 19 days following the first dose of vaccine, 30.0% had anti-S detected in serum which increased to 70.2% at days 20-39. In the 19 days following a second dose, anti-S positivity was 84.2% (32/38). However, pseudovirus neutralization titers (pVNT80) decreased from days 20 to 39.

**Conclusion:**

Despite the immunosuppressive effects of chemotherapy, 2 doses of SARS-CoV-2 vaccines are able to elicit a protective immune response in patients’ ongoing treatment for gastrointestinal cancers. Decreases in pseudoviral neutralization were observed after 20-39 days, re-affirming the current recommendation for vaccine booster doses.

**Clinical Trial Registration Number:**

NCT04427280.

## Teaser

Evidence shows that patients with cancer are at a high risk of severe complications and poor outcomes from SARS-CoV-2 infection. The CARDS study assessed SARS-CoV-2 immunity in patients with gastrointestinal cancer receiving anti-cancer therapy.

**Implications for Practice**

The CARDS study constitutes, to the authors’ knowledge, the largest cohort of patients with gastrointestinal cancer ongoing a primary course of SARS-CoV-2 vaccination. Patients with gastrointestinal cancers undergoing systemic anti-cancer therapies are able to mount immune responses to SARS-CoV-2 vaccines. This provides reassurance to clinicians and patients when considering chemotherapy treatments during the COVID-19 pandemic. Loss of effectiveness of vaccines is evident as early as 20–39 days following the 2nd dose and booster vaccine doses are recommended.

## Introduction

Globally, COVID-19 caused by the SARS-CoV-2 virus has led to over 440 million infections and approximately 6 million deaths to date.^1^ There is substantial evidence patients with cancer are at a high risk of severe complications and poor outcomes from SARS-CoV-2 infection.^2,3^

It is unclear if an immunity to COVID-19 is maintained during chemotherapy and if patients undergoing cytotoxic chemotherapy are able to mount protective immune responses to SARS-CoV-2. These factors have important consequences for the health of patients and the control of SARS-CoV-2 transmission within healthcare facilities emphasizing, the need to establish the effectiveness of SARS-CoV-2 vaccines in patients with cancer.

The Pfizer/BioNTech BNT162b2 and Oxford-AstraZeneca ChAdOx1 nCOV-19 vaccines induce immune responses to the SARS-CoV-2 spike protein and are highly effective in preventing severe complication and death from COVID-19.^4,5^ As patients with malignancy were excluded from these vaccine trials, there is no randomized data which characterizes these vaccines’ efficacies in populations receiving immunosuppressive anti-cancer therapy.

The prioritization of high first dose uptake, extended dosing intervals in the United Kingdom to a maximum of 12 weeks rather than 3-6 weeks as recommended by the vaccine manufacturers.^4,6^ The effect of this off-label dosing in cancer patients is unclear. Worldwide, gastrointestinal malignancies including colorectal, oesophagogastric, and hepato pancreatic and biliary cancers are a leading cause of cancer-related mortality.^7^ Cohort studies to date have reported seroconversion following two doses of mRNA SARS-CoV-2 vaccines in patients with cancer; however, the magnitude of serological responses was lower compared with healthy control groups.^8-10^ To date, there is a paucity of data reporting the immunogenicity of SARS-CoV-2 vaccines, specifically in patients with gastrointestinal malignancies. To address these concerns, we conducted the CARDS (Cancer: Rapid Diagnostics and Immune assessment for SARS-CoV-2) study to assess the immune status of SARS-CoV-2 immunity in gastrointestinal cancer patients who are receiving anti-cancer therapy.

## Materials and Methods

### Study Protocol

The study enrolled patients aged ≥18 years with early or advanced/metastatic malignancy receiving or planning to receive radiotherapy, systemic chemotherapy, or targeted therapy. Eligible patients had no symptoms of acute SARS-CoV-2 infection at enrolment. There were no exclusion criteria. Prior to any study specific procedures, all patients provided voluntary written consent. Enrolled patients were scheduled to have blood taken (serum and EDTA whole blood) at baseline (day 0), day 28, day 56, and day 84. In line with hospital SARS-CoV-2 socially distanced infection control measures, blood tests were scheduled at the time of clinical assessment prior to, or at the time of, anti-cancer therapy administration. As part of the standard of care, patients received either the Pfizer/BioNtech BNT162b2 or the Oxford/AstraZeneca ChAdOx1 vaccine with a maximum interval of 12 weeks between the first and second doses.^6^ Patients were advised to receive a vaccination when invited by local authorities on days when concomitant anti-cancer therapy was not administered.

The primary endpoint was the proportion of patients with a positive detection of (i) anti-nucleocapsid antibodies (anti-NP), and (ii) anti-spike antibodies (anti-S) at each sample timepoint (D0, D28, D56, and D84). The secondary endpoints were the proportion of patients with a positive detection of (i) anti-NP, and (ii) anti-S amongst vaccinated and unvaccinated participants at each timepoint.

The CARDS study was approved by the Newcastle and North Tyneside Research Ethics Committee, United Kingdom (20/NE/0139).

### Assays

Serum SARS-CoV-2 S1 RBD Spike antibodies (anti-S) were measured using the COV2T assay on an Atellica analyser (Siemens). Index values ≥1.0 were considered positive as per the manufacturer’s protocol. Nucleocapsid (anti-N) antibodies were analyzed with the Elecsys SARS-CoV-2 assay on a Cobas analyser (Roche). As specified by the manufacturer, values above a cut-off index (COI) ≥ 1.0 were reported as positive.

Pseudovirus containing wild-type SARS-CoV-2 spike protein was generated in HEK293T cells transfected with p8.91 (packaging), pCSFLW (reporter: luciferase) and pCAGGS-SARS-CoV-2 Spike.^11^ The pseudovirus was collected from culture supernatant and titrated with HEK 293T ACE2-TMPRSS2 (HEK293T-AT) expressing cells (Genecopia, SL222) to determine the dilution required to achieve 2 × 10^6^ relative light units (RLU)/mL.

In a 96-well white plate (Grenier Bio-One, Germany), patient serum was diluted 1/20 in DMEM/2% fetal calf serum (FCS)/penicillin-streptomycin, in duplicate, and then combined with an equal volume of pseudovirus in DMEM/2% FCS/penicillin-streptomycin (final serum dilution, 1/40) and incubated for 1 h at 37 °C. Following this incubation, 10 000 HEK293T-AT cells were added to each well. Controls: negative control (cells only), positive control (known neutralizing serum, diluted at 1/80), and a maximum luciferase control (pseudovirus with cells, no serum). The plate was then incubated for 48 h at 37 °C 5% CO_2_. At 48 h the supernatant was removed from each well. Bright-Glo™ luciferase substrate solution (Promega, Madison, WI, USA) diluted 1:1 with PBS was added and read on a luminescence reader.

All wells were normalized to the maximum luminescence control and samples that had an average of 50% or more suppression of luminescence (pVNT50) were deemed neutralizing, allowing the capture of a wide range of responses, while reducing false positives.^12,13^ For serum samples that were pVNT80 positive at 1 in 40, a further dilution series was carried out. Starting at 1 in 40 and then 2-fold serial dilutions 8 times, in columns, to 1 in 5120 in a 96-well white plate. The dilution at which the sample still achieved pVNT80 was recorded.

### Statistical Analysis

All patients who provided at least 1 blood sample were included in the analysis. All analyses were performed in STATA (v17.0), including the calculation of 95% CIs for proportions and the creation of all plots. The CONSORT diagram was created in Microsoft Visio.

### Role of the Funding Source

The funder of the study had no role in the study design, data collection, data analysis, data interpretation, or writing of the manuscript. D.L., A.T., M.A., and S.R. had full access to all the data. S.R. had final responsibility for the decision to submit for publication.

## Results

### Patient Characteristics

**Figure 1.**
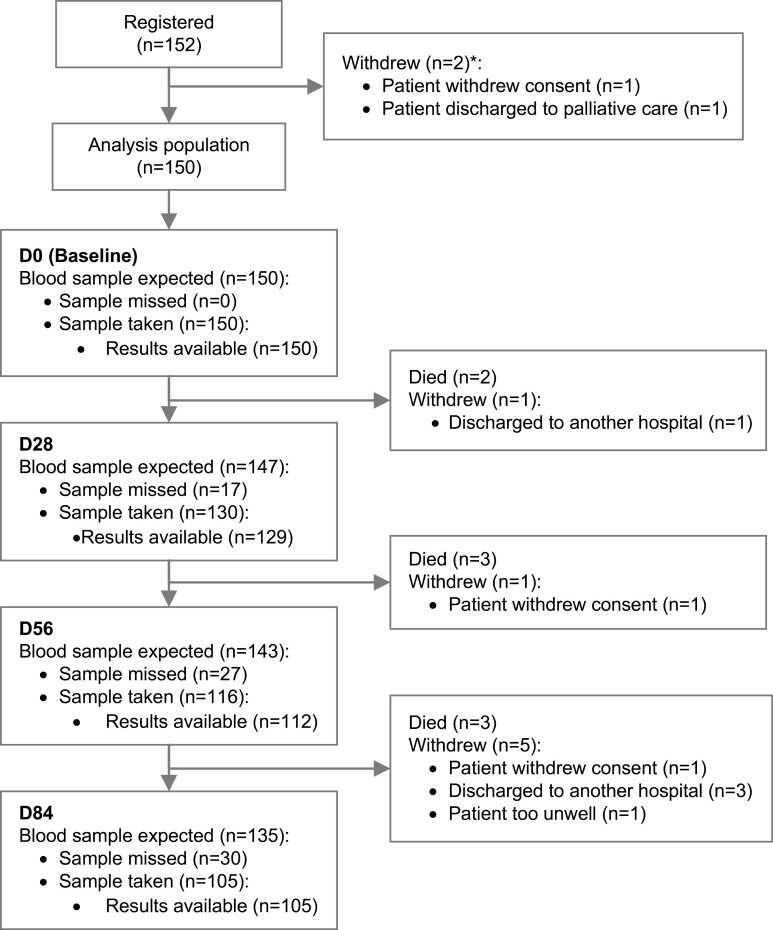
CONSORT diagram of the CARDS study. *Patients RM5287107, RF5287069 withdrew before blood samples were taken. Abbreviations: D0, baseline; D28, day 28; D56, day 56; D84, day 84.

Between September 2020 and April 2021 which was predominantly during the peak of the Delta variant of concern, 152 patients undergoing chemotherapy at the Royal Marsden Hospital, London, United Kingdom were recruited to the study. Of these, 17 patients died or withdrew from the study including 2 patients who were replaced prior to the collection of any blood samples. Across all timepoints, 501 blood samples were taken with results available for 496 blood samples (Fig. 1).

Of the 152 patients, 60 (39.5%) were female. The median age was 66 years (range 30-89 years). Participants were predominantly Caucasian (84.2%). The majority of patients were undergoing chemotherapy for colorectal (*n* = 80, 52.6%), oesophagogastric (*n* = 38, 25.0%), or hepato pancreatic and biliary (*n* = 19, 12.5%) malignancies. A total of 35.5% of patients had an early-stage malignancy, whilst 64.5% had an advanced stage. The most common medical co-morbidities were cardiovascular disease (34.9%), diabetes (17.1%), and previous venous thromboembolism (6.6%).

Most patients were receiving systemic anti-cancer therapy (*n* = 151, 99.3%) most often, a doublet regimen (*n* = 81, 53.9%), followed by chemotherapy single agent chemotherapy (*n* = 18, 11.8%) or triplet regimens (*n* = 14, 9.2%). Immune checkpoint anti-PD1/PD-L1 therapy was administered to 22 patients (14.5%) of whom 9 patients (5.9%) were receiving in combination with chemotherapy. Chemoradiotherapy was administered to 9 patients (5.9%) (Table 1).

**Table 1.** Baseline patient characteristics.

Charateristic	*N* (total = 152)	%
Sex		
Female	60	39.5
Male	92	60.5
Median age, years (IQR 25-75%)	66 (58-72)	
Ethnicity		
Caucasian	128	84.2
Mixed race	1	0.7
Asian	10	6.6
African	2	1.3
Caribbean	3	2.0
Oriental	2	1.3
Other	6	4.0
Anatomical site of malignancy		
Colorectal	80	52.6
Oesophagogastric	38	25.0
Hepato pancreatic and biliary	24	15.8
Other^*^	10	6.6
Cancer stage		
Early	54	35.5
Advanced	98	64.5
Comorbidities		
Cardiovascular disease^**^	53	34.9
Diabetes	26	17.1
Venous thromboembolism	10	6.6
Asthma/COPD	10	3.9
Chronic liver disease	4	2.6
Autoimmune disorder	3	2.0
Obesity	2	1.3
Chronic kidney disease	1	0.7
Current anticancer therapy		
Systemic therapy	151	99.3
Chemotherapy singlet	18	11.8
Chemotherapy doublet	81	53.9
Chemotherapy doublet + anti PD1/PDL1	2	1.3
Chemotherapy triplet	14	9.2
Chemotherapy triplet + anti PD1/PDL1	7	4.6
Chemotherapy with radiotherapy	9	5.9
Anti-PD1/PDL1	13	8.6
Other targeted therapy^***^	7	4.6

Includes 3 patients with Non-Hodgkin Lymphoma, 3 patients with carcinoma of unknown primary, 2 patients with anal cancer, and 1 patient each with appendiceal cancer and neuroendocrine carcinoma.

Includes hypertension, heart failure, ischaemic heart disease, and cerebrovascular disease.

Includes 2 patients receiving olaparib and 1 patient each receiving lanreotide, derazantinib, ramucirumab, rituximab, trastuzumab deruxtecan.

### Primary Outcome

Anti-S antibodies were detected at D0, D28, D56, and D84 in 34.9% (95% CI, 27.2-43.3), 38.3% (29.8-47.3), 52.7% (43.0-62.2), and 61.9% (51.9-71.2) of participants respectively. A total of 23 patients (15.3%) had anti-NP positivity at any point during the study (Supplementary Table S1).

### Unvaccinated Patients

SARS-CoV-2 vaccines were available from December 2020. Fifty-one participants did not receive a vaccine dose prior to, or during the study. Five patients (9.8%) had detectable anti-NP and anti-spike antibodies during the course of the study which was due to prior COVID-19 infection (Supplementary Table S2).

### Vaccinated Cohort

SARS-CoV-2 vaccines were available in the United Kingdom from December 2020. Ninety-nine patients received at least 1 dose of a vaccine prior to enrolment or during the study. Forty-six patients (46%) received Pfizer BioNtech BNT162b2 and 50 patients (51%) received the Oxford/AstraZeneca ChAdOx1 vaccine whilst in 3 patients (3.0%) the vaccine received was undetermined. The median duration between the first and second dose received was 11.0 weeks (IQR 9.5, 11.7).

Prior to study entry, 64 patients received at least 1 vaccine dose. The proportion of anti-S antibody positivity amongst these patients was 61.9% (95% CI, 48.8-73.9), 71.7% (57.7-83.2), 81.3% (67.4-91.1) and 91.5% (79.6-97.6) at D0, D28, D56, and D84, respectively.

Next, we determined neutralizing antibody activity by a pseudovirus assay which relies upon replication-defective viral particles expressing the “wild-type” SARS-CoV-2 Spike protein^11^ infecting HEK293T ACE2-TMPRSS2 expressing cells. The corresponding prevalence of positive neutralizing antibodies (achieving pVNT50 at 1/40 serum dilution) were similarly high at D0 (59.4%, 95% CI, 46.4-71.5), D28 (67.9%, 53.7-80.1), D56 60.0% (45.2-73.6), and at D84 (83.0%, 69.2-92.4) (Table 2).

**Table 2.** Prevalence of anti-spike antibodies and pseudovirus neutralisation in patients who had received one dose of vaccine prior to enrolment.

Timepoint	Anti-S		Pseudovirus neutralisation (1/40 titre)	
	Positive *n* (%)	95% CI	Positive^*^ n (%)	95% CI
D0	39/63 (61.9)	48.8-73.9	38/64 (59.4)	46.4-71.5
D28	38/53 (71.7)	57.7-83.2	36/53 (67.9)	53.7-80.1
D56	39/48 (81.3)	67.4-91.1	30/50 (60.0)	45.2-73.6
D84	43/47 (91.5)	79.6-97.6	39/47 (83.0)	69.2-92.4

Positive pseudovirus neutralisation defined as inhibition >0.5 (pVNT50) at 1/40 dilution.

Abbreviation: anti-S, anti-spike.

As part of a sensitivity analysis, we excluded patients with positive anti-NP as the previous infection confers prolonged protective antibody responses.^14^ The positivity rates of anti-spike and neutralizing antibodies were similarly high (Supplementary Table S3).

**Figure 2.**
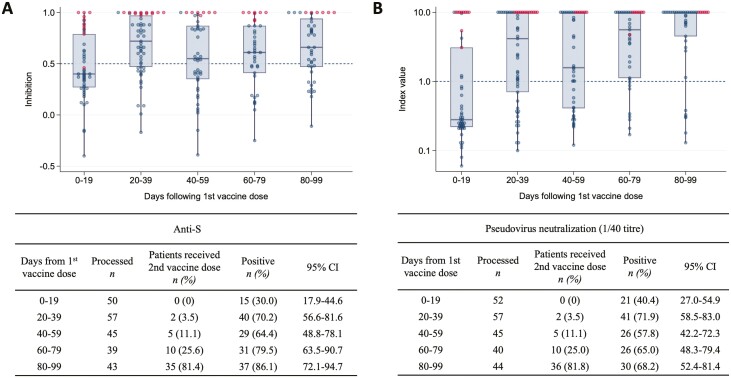
Longitudinal analysis of SARS-CoV-2 vaccine immunogenicity relative to the 1st vaccine dose. Box plot of Anti-S index values (**A**) and pseudovirus neutralisation (titre 1/40) in all vaccinated patients (**B**) at time periods relative to the date of 1st vaccine dose. Each data point represents a serum sample. Solid horizontal lines denote the median and boxes represent the interquartile range. Samples from patients with positive nucleocapsid results are marked in red and negative in blue. Positivity thresholds are denoted by dotted lines (anti-S > 1.0, pVNT(1/40) > 0.5). Abbreviation: anti-S, anti-spike antibody.

To further assess the longitudinal evolution of anti-S antibody responses following vaccination, we analyzed the vaccine cohort from the date of the first vaccine. Within the first 19 days following the 1st dose of SARS-CoV-2 vaccination, 30.0% (95% CI, 17.9-44.6) had anti-S detected in serum with 40.4% (27.0-54.9) having neutralizing antibodies. By days 20-39 this had increased to 70.2% (56.6-81.6) and 71.9% (58.5-83.0), respectively. Consistent with previous reports following single-dose vaccination,^15^ there was a plateau in seropositivity at days 40-59 (64.4%) and neutralizing antibody activity also mirrored this trend (Fig. 2). The majority of patients with previous COVID-19 infection had high anti-S antibody levels and pseudoviral neutralization activity.

**Figure 3.**
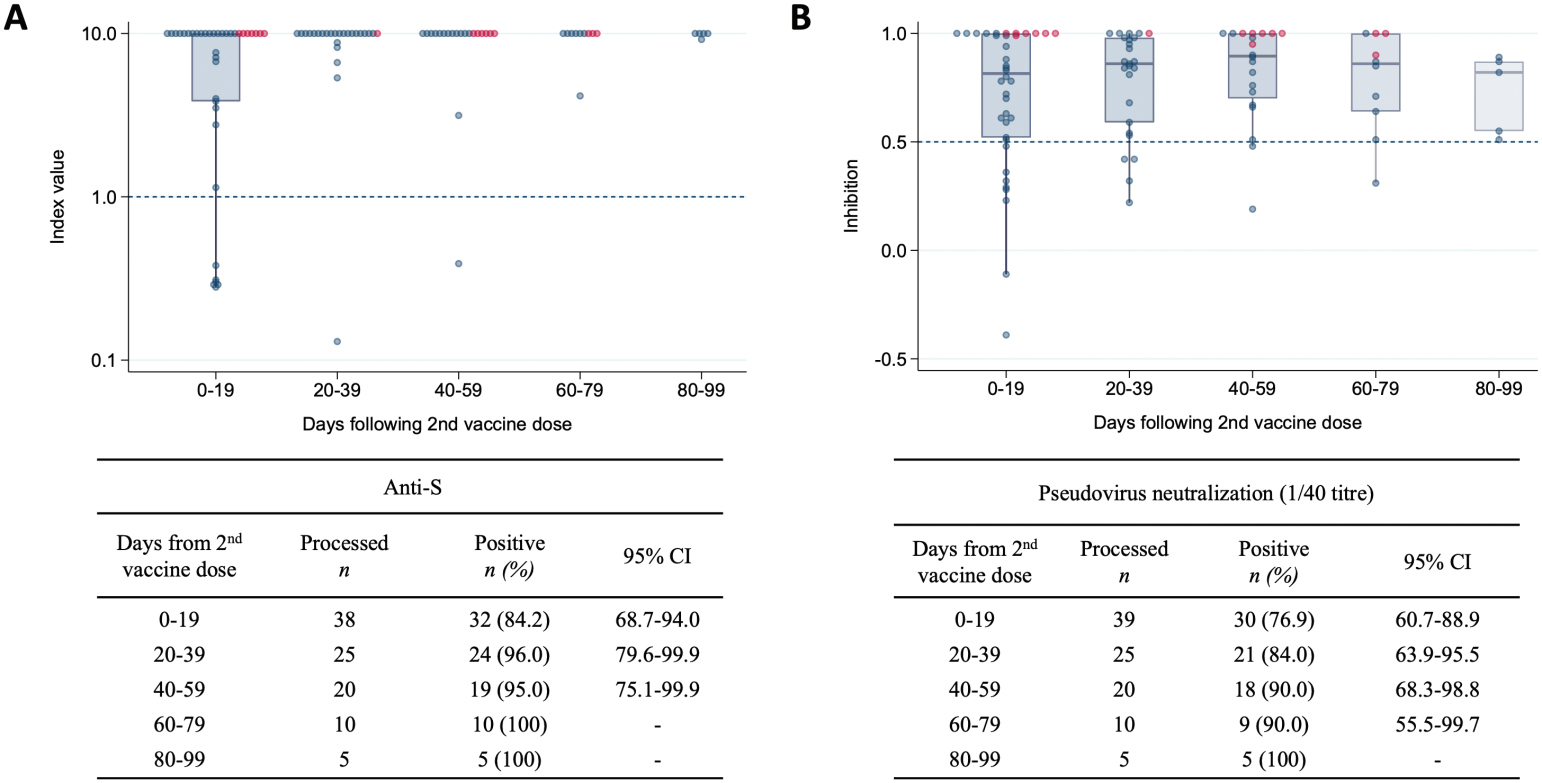
Longitudinal analysis of SARS-CoV-2 vaccine immunogenicity following 2nd vaccine dose. Box plot of anti-S index values (**A**) and pseudovirus neutralisation (titre 1/40) in all vaccinated patients (**B**) at time periods relative to the date of 2nd SARS-CoV-2 vaccine dose. Each data point represents a serum sample. Solid lines denote the median and boxes represent the interquartile range. Samples from patients with positive nucleocapsid results are marked in red and negative in blue. Prespecified positivity thresholds are denoted by dotted lines (anti-S > 1.0, anti-S > 1.0, pVNT(1/40) > 0.5).

Within the 19 days following a second dose of SARS-CoV-2 vaccination, anti-S positivity and viral neutralization was 84.2% (68.7-94.0) and 76.9% (60.7-88.9), respectively. At 40-59 days, seropositivity and viral pseudoviral neutralization were 95.0% (75.1-99.9), and 90.0% (68.3-98.8) (Fig. 3). Though numbers are smaller, at days 60-79 and 80-99, the majority remained seropositive with pseudovirus neutralization. Two patients with non-Hodgkin lymphoma ongoing anti-CD20 therapy had no detectable anti-S or pseudoviral neutralization at any timepoint following the second vaccine dose.

**Figure 4.**
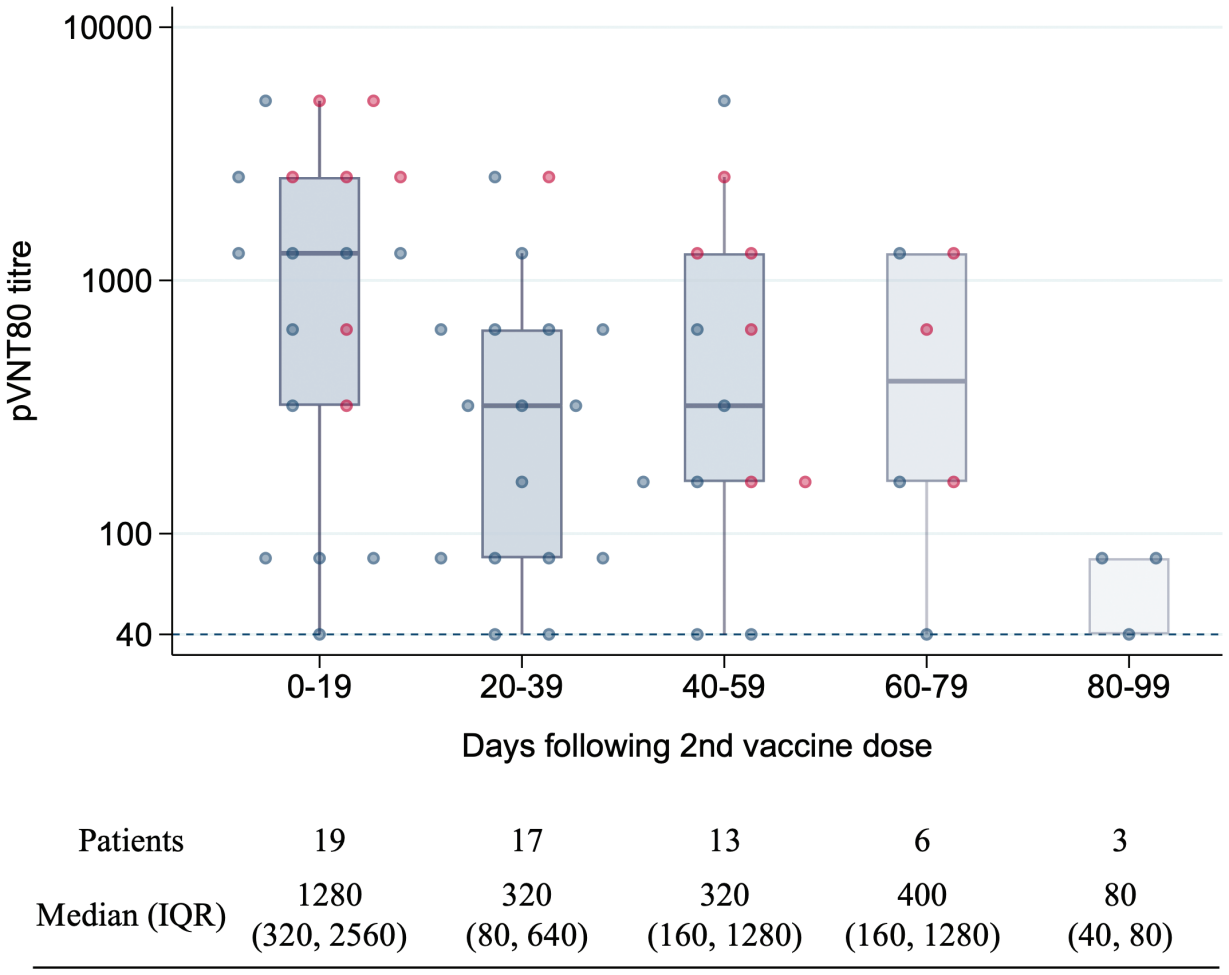
Box plots of pVNT80 neutralization titers after the 2nd dose of SARS-CoV-2 vaccine. A logarithmic scale was used for neutralization titre. Each data point represents a serum sample. Solid lines denote the median and boxes represent the interquartile range. Samples from patients with positive nucleocapsid results are marked in red and negative in blue.

To further understand the magnitude of humoral response following the second dose of SARS-CoV-2 vaccine, we ascertained the pVNT80 dilution titre for blood samples with inhibition of 0.8 at the 1/40 dilution (Fig. 4). At day 0-19, the median pVNT80 titre was 1/1280 (IQR 1/320-1/2560). At the days 20-39, 40-59, and 60-79 timepoints the dilution titre had decreased to 1/320 (1/80-1/640), 1/320 (1/160-1/1280), and 1/400 (1/160-1/1280), respectively.

### Sensitivity/Specificity

To validate the performance of the Atellica COV2T assay in assessing vaccine responses we excluded patients with positive anti-NP antibodies. In comparison to pseudovirus neutralization, the sensitivity, and specificity of anti-S antibody detection were 80.6%, and 95.7%, respectively (D0). Across all time points, the sensitivity and specificity were similarly high (Supplementary Table S4).

## Discussion

To our knowledge, this is the largest, prospective study of a cohort of gastrointestinal cancer patients receiving anti-cancer treatment to have characterized the response to SARS-CoV-2 vaccination. We demonstrated patients were able to mount humoral immune responses to SARS-CoV-2 vaccines and that the immunological responses to vaccination were maintained in patients who had received 1 dose of vaccine prior to systemic cancer therapy initiation. Following a second dose of vaccination, anti-S antibody and pseudovirus neutralization positivity were high and in keeping with previous reports in other solid tumor cohorts.^8,10,16-19^

Two patients with non-Hodgkin lymphoma receiving anti-CD20 therapy did not have anti-S or pseudovirus neutralizing antibodies detectable after 2 doses of vaccination. Previous reports have confirmed even poorer seroconversion rates in patients with hematological malignancies including acute leukaemia,^20^ multiple myeloma,^21^ chronic lymphocytic leukaemia,^22,23^ and lymphomas particularly in patients receiving B-cell depleting therapies such as anti-CD20 therapy.^24^ This highlights the need for booster vaccinations and non-pharmacological interventions to prevent SARS-CoV-2 infection in patients with hematological malignancy.

In the United Kingdom, the duration between the first and second doses of vaccination was extended from 3 weeks to 12 weeks after reports of higher vaccine efficacy and to increase vaccine availability to the wider public.^6^ After 40-59 days following the first dose, we observed a small drop in detectable anti-S antibodies which was abrogated by the administration of a second vaccine dose. Whilst this observation may provide an argument to shorten the dosing interval, the low number of infections is also evidence of the effectiveness of other measures such as social distancing, hygiene practices, and hospital infection control policies. This study was conducted during the peak of the Delta (B.1.617.2) variant outbreak in the United Kingdom and these non-pharmaceutical interventions are recommended particularly during outbreaks of highly infectious SARS-CoV-2 variants such as Omicron (B.1.1.529).

Our data suggest a decrease in pseudovirus neutralization titers after 20-39 days following the second dose of SARS-CoV-2 vaccination. Due to the length of follow-up in this study and the size of the sample set, we were not able to definitively assess the duration of vaccine immunogenicity or the factors underpinning this observation. Nevertheless, this result is in keeping with recent reports which have confirmed serological responses to SARS-CoV-2 vaccines significantly wane after 6 months in healthy subjects.^14^

We validated the use of qualitative anti-S antibody measurement as a surrogate for SARS-CoV-2 immunity. At the manufacturers’ recommendation of an anti-S antibody positivity cut-off of >1.00, high rates of sensitivity and specificity were observed when compared with in vitro pseudovirus neutralization. International standardization of anti-S antibody measurements and neutralization assays is underway which will be useful in determining the value of using serum antibodies as a surrogate for vaccine effectiveness.^25-27^

Our study is not without its limitations. One of the caveats to our study is the inevitable rates of attrition and study compliance which largely occurred due to constrains on hospital visits; hence, selection bias should be considered when interpreting these data.

This is particularly pertinent given patients with solid organ malignancies mount lower anti-S antibody titers compared to healthy subjects. Whilst we did not measure the level of SARS-CoV-2 immunity in relation to a control cohort, we would expect immune responses to be attenuated, given previous reports.^10,28^

Our pseudovirus neutralization assays were modeled upon the Wuhan strain SARS-CoV-2 Spike protein, and viral neutralization to other variants of concern (VOC) was not assessed. Whilst recent studies have observed significant immune escape with the Delta and Omicron variants,^29,30^ vaccine booster doses based upon the first wave virus are effective against these VOCs. Given the waning in vaccine immunity and the emergence of VOCs, booster doses are now widely recommended.^31^

Serological responses are only part of the protective immune response and whilst we did not assess other mechanisms of SARS-CoV-2 cell-mediated immunity measurable in peripheral blood such as T-cell immunity. Previous studies have reported serological assays and virus neutralization are well-correlated following natural infection^25^ and correlate with vaccine effectiveness.^32^ It is reassuring that previous studies have been in line with our observations and also report that T-cell responses are maintained following SARS-CoV-2 vaccination in patients with solid organ malignancy.^16^

In summary, we have demonstrated that in gastrointestinal cancer patients undergoing chemotherapy, recipients of SARS-CoV-2 vaccination are able to mount immunological responses to a primary course of SARS-CoV-2 vaccination. Whilst these data should provide reassurance to patients with cancer and for clinicians when deciding upon cancer treatments during the COVID-19 pandemic, the duration of humoral responses is likely to be limited and booster doses are recommended.

## Supplementary Material

oyac230_suppl_Supplementary_Material

## Data Availability

The data underlying this article will be shared on reasonable request to the corresponding author.
