# Supplementary material for: SARS-CoV-2 Vaccine Immunogenicity in Patients with Gastrointestinal Cancer Receiving Systemic Anti-Cancer Therapy
Source: Oncologist. 2022 Nov 7;28(1):e1–8. doi: 10.1093/oncolo/oyac230 (PMC9847553; doi:10.1093/oncolo/oyac230)
Supplement: oyac230_suppl_Supplementary_Material [file oyac230_suppl_supplementary_material.docx]

Supplementary Table 1: Prevalence of anti-nucleocapsid and anti-spike antibodies in the entire population at each timepoint

|  |  | Anti-NP | | Anti-S | |
| --- | --- | --- | --- | --- | --- |
| Timepoint | Blood Collected, n | Positive,  n (%) | 95% CI | Positive, n (%) | 95% CI |
| D0 | 150 | 22/150 (14·7) | 9·4-21·4 | 51/146 (34·9) | 27·2-43·3 |
| D28 | 130 | 18/129 (14·0) | 8·5-21·2 | 49/128 (38·3) | 29·8-47·3 |
| D56 | 116 | 18/112 (16·1) | 9·8-24·2 | 59/112 (52·7) | 43·0-62·2 |
| D84 | 105 | 15/105 (14·3) | 8·2-22·5 | 65/105 (61·9) | 51·9-71·2 |

Abbreviations: NP – nucleocapsid, S – spike, CI – confidence interval

Supplementary Table 2: Prevalence of anti-nucleocapsid and anti-spike antibodies in the unvaccinated population

|  | Anti-NP^1^ | | Anti-S | |
| --- | --- | --- | --- | --- |
| Timepoint | Positive,  n (%) | 95% CI | Positive, n (%) | 95% CI |
| D0 | 4/51 (7·8) | 2·2-18·9 | 4/49 (8·2) | 2·3-19·6 |
| D28 | 3/44 (6·8) | 1·4-18·7 | 3/43 (7·0) | 1·5-19·1 |
| D56 | 4/34 (11·8) | 3·3-27·5 | 5/34 (14·7) | 5·0-31·1 |
| D84 | 1/27 (3·7) | 0·0-19·0 | 1/27 (3·7) | 0·0-19·0 |

^1^ 5 patients presented a positive nucleocapsid result at any given timepoint

Abbreviations: NP – nucleocapsid, S – spike, CI – confidence interval

Supplementary Table 3: Prevalence of anti-spike antibodies and pseudovirus neutralisation in the vaccinated population, including only patients who had not tested positive for anti-nucleocapsid antibodies

|  | Anti-S | | Pseudovirus neutralisation (1/40 titre) | |
| --- | --- | --- | --- | --- |
| Timepoint | Positive,  n (%) | 95% CI | Positive*, n (%) | 95% CI |
| D0 | 28/52 (53·9) | 39·5-67·8 | 28/53 (52·8) | 38·6-66·7 |
| D28 | 29/44 (65·9) | 50·1-79·5 | 27/44 (61·4) | 45·5-75·6 |
| D56 | 31/40 (77·5) | 61·5-89·2 | 22/42 (52·4) | 36·4-68·0 |
| D84 | 35/39 (89·7) | 75·8-97·1 | 31/39 (79·5) | 63·5-90·7 |

* Positive pseudovirus neutralisation defined as inhibition >0.5 (pVNT50) at 1/40 dilution

Abbreviations: Anti-S – anti-spike, CI – confidence interval

Supplementary table 4: Sensitivity and specificity of anti-S antibodies at each blood sample timepoint (D0, D28, D56, D84), in comparison to pseudovirus neutralisation

|  |  | D0,  n=146 | | D28,  n=128 | | D56,  n=112 | | D84,  N=105 | |
| --- | --- | --- | --- | --- | --- | --- | --- | --- | --- |
|  |  | Pos | Neg | Pos | Neg | Pos | Neg | Pos | Neg |
| Anti-S | Pos. | 46 | 5 | 42 | 7 | 44 | 15 | 59 | 6 |
|  | Neg. | 6 | 89 | 10 | 69 | 5 | 48 | 2 | 38 |
| Sensitivity | | 88·5 | | 80·8 | | 89·8 | | 96·7 | |
| Specificity | | 94·7 | | 90·8 | | 76·2 | | 86·4 | |

Positive pseudovirus neutralisation defined as inhibition >0.5 (pVNT50) at 1/40 dilution

Abbreviations: Pos – positive, Neg - negative
